# Prevalence of Diabetes and Hypertension Among King Abdulaziz University Employees: Data From First Aid and Cardiopulmonary Resuscitation Training Program

**DOI:** 10.7759/cureus.20097

**Published:** 2021-12-02

**Authors:** Mawya A Khafaji, Kamal W Al Ghalayini, Maram K Sait, Rafal A Alorri, Tasneem Garoub, Esrra A Alharbi, Talah Magadmi, Falwah Fatani, Hussain F Jan, Abdulkarim A Jawhari

**Affiliations:** 1 Radiology, Faculty of Medicine, King Abdulaziz University, Jeddah, SAU; 2 Medicine, Faculty of Medicine, King Abdulaziz University, Jeddah, SAU; 3 Internal Medicine, King Abdullah Medical City, Jeddah, SAU; 4 Radiology, National Guard Hospital, Jeddah, SAU; 5 Preventive Medicine, King Khalid University Hospital, Jeddah, SAU; 6 Internal Medicine, King Faisal Specialist Hospital and Research Centre, Jeddah, SAU; 7 Internal Medicine, King Abdulaziz University, Jeddah, SAU; 8 Family Medicine, King Abdulaziz University, Jeddah, SAU; 9 Faculty of Medicine, King Abdulaziz University, Jeddah, SAU

**Keywords:** saudi arabia, risk factors, body mass index, diabetes mellitus, hypertension

## Abstract

Objectives

Saudi Arabia has a very high rate of chronic illnesses, especially hypertension (HTN) and diabetes. This study aimed to investigate the prevalence and control of diabetes and hypertension among employees at a university in Saudi Arabia, including the associated risk factors, and to evaluate the need for early screening among these individuals.

Methods

This retrospective study used data from the first aid training program. In total, there were 3964 employees who completed the program, and only 1000 employees were enrolled. The program was conducted at King Abdulaziz University (KAU), Jeddah, Saudi Arabia. Blood pressure (BP), random blood sugar, and body mass index (BMI) were measured in all employees. Descriptive data, including mean, standard deviation (SD), crosstab, chi-square, and linear regression, were analyzed. Categorical variables were described using frequencies and percentages.

Results

The prevalence of hypertension and diabetes was 31% and 5%. There were 365 males and 635 females. Employees with risk factors such as gender, age, and body mass index had significant effects on having high blood pressure and random blood glucose measurements. Of the employees who reported being free from chronic diseases, 2.9% had abnormal random blood glucose readings (prediabetic and diabetic ranges), while 37.4% had abnormal blood pressure readings (prehypertensive and hypertensive ranges).

Conclusion

The high prevalence of hypertension and diabetes reflects the crucial role of early screening in diabetes and hypertension protocols and raising awareness regarding protocol implementation in Saudi Arabia to improve quality of life (QoL) at the individual and community levels.

## Introduction

Diabetes mellitus (DM) is a metabolic disease characterized by elevated levels of blood glucose caused by defects in insulin secretion or insulin resistance [1]. Hypertension (HTN) or increased blood pressure (BP), according to the American College of Cardiology, is defined as systolic blood pressure (SBP) of 130-139 mmHg or diastolic blood pressure (DBP) of 80-98 mmHg for stage 1 HTN or SBP of ≥140 mmHg or DBP of ≥90 mmHg for stage 2 HTN [2].

The prevalence of HTN and DM has been studied in different regions because of the increase in these diseases over time. Low rates of awareness, initiation of treatment, and control of disease progression cause additional burdens to the healthcare system [3]. Saudi Arabia is one of the leading nations worldwide with a high prevalence of chronic diseases and their associated risk factors. One in four adults is either obese or diabetic. The prevalence of HTN in the population is 25% and that of coronary heart disease is nearly 6% [4]. In 1992, approximately 0.9 million people were diagnosed with DM according to a report published by the Saudi Arabia Ministry of Health; however, in less than two decades, there was an increase of 2.7 times the incidence rates, where the figure increased to reach 2.5 million people in 2010 [5].

Nevertheless, some risk factors incline patients to the development of DM [6]. Smoking, a modifiable risk, showed a correlation with DM. The five-year-long Insulin Resistance Atherosclerosis Study considered multiple risk factors such as anthropometric factors [7]. Another modifiable risk is body mass index (BMI), wherein daily routine activities, healthy lifestyle, good-quality food, and physical activity can reduce the probability of developing DM [8]. Aging is also associated with physiological changes that decrease insulin sensitivity. Hence, with increasing age, the prevalence increases [5,8-10]. HTN prevalence has similar modifiable and nonmodifiable risk factors, such as the increasing percentages of individuals with central obesity, increasing age, and smoking [11]. HTN itself is linked to the increasing prevalence of DM [12].

Moreover, a previous study conducted among 19.1 million participants from 1975 to 2015 stated that BP varied during the past four decades, where it became higher in low-income countries due to contrasting trends, while it remained persistently high in Central and Eastern Europe [13].

A systemic analysis attributed the global burden of 87 risk factors. Elevated SBP was found to be the leading cause of death in females and the second most common cause of death in males. DM has a 100% relationship with the risk factor fasting plasma glucose, and it is the third leading cause of death in females and fifth in males [14]. Both DM and HTN are risk factors for cardiovascular disease (CVD), peripheral vascular disease, kidney disease, and other morbidities. CVD is the leading cause of death in DM and a major consequence of HTN [5,15]. DM is the leading contributor to end-stage renal disease [16], while HTN causes renal arteriolar sclerosis and nephrosclerosis, leading to chronic kidney diseases [17]. Strict glycemic control delays the risk of CVD [18]. As for health-related quality of life (HRQoL), a systematic review and meta-analysis conducted in Fortaleza, Brazil, in 2015 validated the correlation between chronic illnesses, specifically HTN, and quality of life in these patients [19]. Likewise, patients with DM, compared to the general population, have a lower score for HRQoL according to a study performed in Saudi Arabia [20].

There is no better way of primary intervention other than screening, and the occurrence of these morbidities could be delayed or even prevented with proper control of the disease and early modifications. Screening is the process of identifying disease in individuals who are unaware of their condition and have more possibilities. They undergo an initial assessment that closely detects the presence of a chronic condition [21]. A cross-sectional study that investigated the awareness of HTN showed that one-third of subjects were unaware of their condition. Another study revealed that people who self-monitor their BP are more aware of how to handle their own readings. Moreover, collaboration with healthcare practitioners and self-monitoring can improve controlling BP [22,23].

Men were found to be at a higher risk of HTN than premenopausal women as proven in recent studies. Although the reason underlying gender differences in HTN remains unknown, there is crucial evidence supported by research conducted among children [24,25]. Another cross-sectional study based in Korea used the data of the National Health Insurance Service-National Sample Cohort (NHIS-NSC) to gain knowledge of how beneficial it is to practice social insurance policies for the families of employees in detecting the prevalence, recognition, and management of chronic diseases including DM, HTN, and dyslipidemia [26].

As we mentioned above, several studies have been conducted globally and in Saudi Arabia specifically, but there is a lack of information and studies about DM and HTN at King Abdulaziz University (KAU). Therefore, this study aimed to measure the prevalence of DM and HTN among employees at King Abdulaziz University, taking into consideration the risk factors, with early screening forming future strategies to prevent and control HTN and DM by identifying and reporting undiagnosed individuals.

## Materials and methods

Ethical approval

Ethical approval was provided by the Biomedical Ethics Research Committee at King Abdulaziz University Hospital (reference number: 441-20).

Study design, sample size, and inclusion criteria

A retrospective study was conducted with a total sample size comprising 1000 staff members of KAU who consented and agreed to participate in the project. All 1000 staff were included as participants, and they were chosen randomly. Students were excluded.

Setting

This study used data from 2018 to 2019, which was conducted in the university campus at Jeddah, Saudi Arabia, between October 28, 2018, and November 27, 2019. KAU is a public university that was established in 1967 and is currently ranked first at many regional ranks and top 200th university in five global rankings.

Program objective and data collection

The training program started in 2018 and aimed to teach cardiopulmonary resuscitation to employees from different colleges across the university campus. The workshop included a screening part where blood pressure (BP), blood glucose, weight, and height were measured, and medical history was taken. The components of the medical history were history of smoking, history of chronic diseases, history of heart disease, and family history of heart diseases or sudden deaths.

BP was measured using a cuff sphygmomanometer (Omron M3, Omron Corporation, Kyoto, Japan). Random blood glucose readings were measured using a device (Contour TS) and thin lancets. BMI was calculated using the following formula: weight (kg)/height (m^2^).

We defined SBP of 130-139 mmHg or DBP of 80-98 mmHg as pre-HTN or SBP of ≥140 mmHg or DBP of ≥90 mmHg as HTN and less than 7.8 mmol/L as normal blood glucose, 7.8-11 mmol/L as prediabetes, and above 11 mmol/L as diabetic.

Statistical analysis

Statistical analysis was performed using the Statistical Package for the Social Sciences (SPSS), version 25, software package (IBM Inc., USA). Continuous variables were presented as mean and standard deviation (SD). Differences between groups were compared using crosstabs, chi-square, and linear regression. Categorical variables were described as frequencies and percentages. The P-value was considered statistically significant at <0.05.

## Results

**Figure 1:**
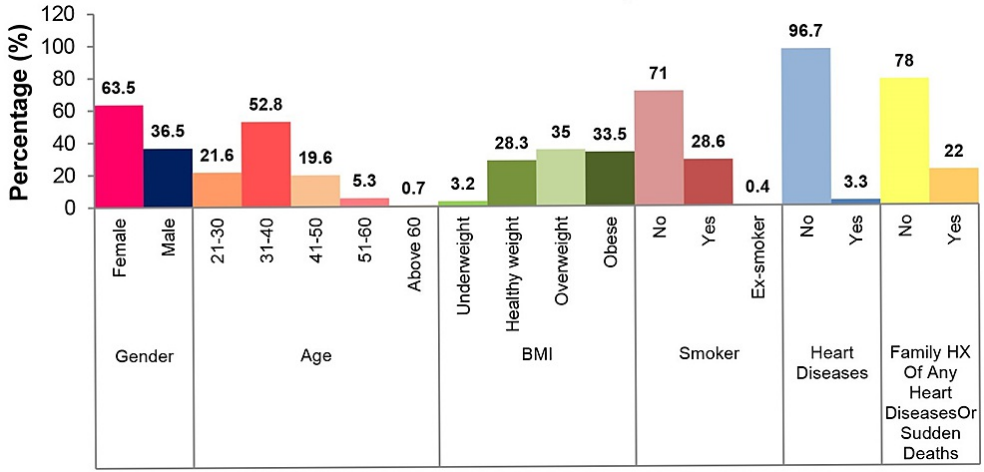
Employees’ data

A total sample size of 1000 employees was included in the study. Figure 1 shows the employees’ data. Overall, 63.5% of the participants were female, while 36.5% were male. The most common age group was between 31 and 40 years old, accounting for 52.8% of the total participants. Regarding BMI, approximately 35% were found to be overweight and 33.5% were obese. Moreover, 28.6% of the employees were current smokers, 3.3% had known heart disease, and 22% had a family history of heart disease or sudden death.

**Figure 2:**
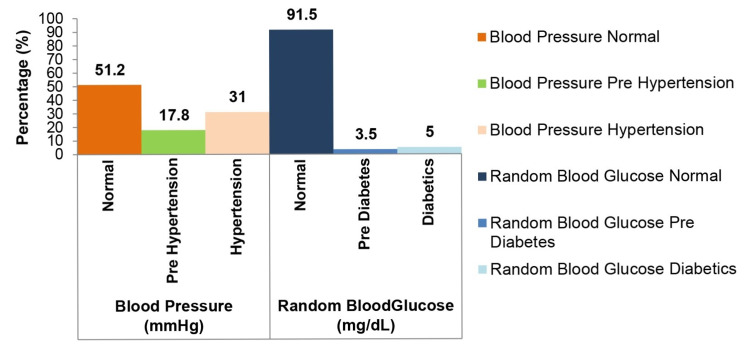
Overall prevalence of the categories of blood pressure and random blood glucose

Figure 2 shows the overall prevalence of the categories of BP and random blood glucose. Concerning BP, 17.8% were prehypertensive and 31.0% had HTN. As for the random blood glucose, only 3.5% were prediabetic and 5.0% were diabetic.

Table 1 and Table 2 show the prevalence of the subdivisions of BP and random blood glucose, the associated risk factors, and their significance.

**Table 1:** Blood pressure ranges with risk factors BMI, body mass index; HTN, hypertension; DM, diabetes mellitus

	Normal	Prehypertension	Hypertension	P-value
	(n = 512)	(n = 178)	(n = 310)	
Gender				
Female	419 (81.8%)	68 (48.3%)	130 (41.9%)	0.000
Male	93 (18.2%)	92 (51.7%)	180 (58.1%)	Significant
Age (years)				
21–30	136 (26.6%)	32 (18%)	48 (15.5%)	
31–40	276 (53.9%)	100 (56.2%)	152 (49%)	0.000
41–50	88 (17.2%)	31 (17.4%)	77 (24.8%)	Significant
51–60	10 (2%)	13 (7.3%)	30 (9.7%)	
Above 60	2 (0.4%)	2 (1.1%)	3 (1%)	
BMI				
Underweight	26 (5.1%)	3 (1.7%)	3 (1%)	
Healthy weight	183 (35.9%)	52 (29.2%)	48 (15.5%)	0.000
Overweight	185 (36.3%)	58 (32.6%)	107 (34.5%)	Significant
Obese	118 (23.1%)	65 (36.5%)	152 (49%)	
Smoker				
(Cigarette – shisha)				
No	387 (75.6%)	113 (63.5%)	210 (67.7%)	0.010
Yes	123 (24%)	65 (36.5%)	98 (31.6%)	Significant
Ex-smoker	2 (0.4%)	0 (0%)	2 (0.6%)	
Chronic diseases				
(None)				
No	54 (10.5%)	27 (15.2%)	87 (28.1%)	0.000
Yes	458 (89.5%)	151 (84.8%)	223 (71.9%)	Significant
(HTN)				
No	496 (96.9%)	167 (93.8%)	267 (86.1%)	0.000
Yes	16 (3.1%)	11 (6.2%)	43 (13.9%)	Significant
(DM)				
No	495 (96.7%)	163 (91.6%)	273 (88.1%)	0.000
Yes	17 (3.3%)	15 (8.4%)	37 (11.9%)	Significant
(Dyslipidemia)				
No	485 (94.7%)	165 (92.7%)	287 (92.6%)	0.391
Yes	27 (5.3%)	13 (7.3%)	23 (7.4%)	Not significant
Heart diseases				
No	495 (96.7%)	175 (98.3%)	297 (95.8%)	0.328
Yes	17 (3.3%)	3 (1.7%)	13 (4.2%)	Not significant
Family history of heart diseases or sudden deaths				
No	391 (76.4%)	139 (87.1%)	250 (80.6%)	0.357
Yes	121 (23.6%)	39 (21.9%)	60 (19.4%)	Not significant

**Table 2:** Random blood sugar ranges with risk factors BMI, body mass index; HTN, hypertension; DM, diabetes mellitus

	Normal	Prediabetes	Diabetes	P-value
	(n = 915)	(n = 35)	(n = 50)	
Gender				
Female	588 (64.3%)	21 (60%)	26 (52%)	0.195
Male	327 (35.7%)	14 (40%)	24 (48%)	Not significant
Age (years)				
21–30	211 (23.1%)	2 (5.7%)	3 (6%)	
31–40	499 (54.5%)	14 (40%)	15 (30%)	0.000
41–50	163 (17.8%)	12 (34.3%)	21 (42%)	Significant
51–60	37 (4%)	5 (14.3%)	11 (22%)	
Above 60	5 (0.5%)	2 (5.7%)	0 (0%)	
BMI (kg/m^2^)				
Underweight	31 (3.4%)	0 (0%)	1 (2%)	
Healthy weight	276 (30.2%)	2 (5.7%)	5 (10%)	0.000
Overweight	320 (35%)	10 (28.6%)	20 (40%)	Significant
Obese	288 (31.5%)	23 (65.7%)	24 (48%)	
Smoker				
(Cigarette – shisha)				
No	647 (70.7%)	29 (82.9%)	34 (68%)	0.563
Yes	264 (28.9%)	6 (17.1%)	16 (32%)	Not significant
Ex-smoker	4 (0.4%)	0 (0%)	0 (0%)	
Chronic diseases				
(None)				
No	112 (12.2%)	17 (48.6%)	39 (78%)	0.000
Yes	803 (87.8%)	18 (51.4%)	11 (22%)	Significant
(HTN)				
No	860 (94%)	29 (82.9%)	41 (82%)	0.000
Yes	55 (6%)	6 (17.1%)	9 (18%)	Significant
(DM)				
No	890 (97.3%)	21 (60%)	20 (40%)	0.000
Yes	25 (2.7%)	14 (40%)	30 (60%)	Significant
(Dyslipidemia)				
No	861 (94.1)	30 (85.7%)	46 (92%)	0.118
Yes	54 (5.9%)	5 (14.3%)	4 (8%)	Not significant
Heart diseases				
No	886 (96.8%)	34 (97.1%)	47 (94%)	0.545
Yes	29 (3.2%)	1 (2.9%)	3 (6%)	Not significant
Family history of heart diseases or sudden deaths				
No	711 (77.7%)	30 (85.7%)	39 (78%)	0.533
Yes	204 (22.3%)	5 (14.3%)	11 (22%)	Not significant

As shown in Table 1, there was a significant difference (P < 0.05) between BP and each of gender, age, BMI, smoking, chronic diseases, HTN, and DM. In other words, these factors had a significant effect on BP. Out of the 1000 participants, 17.8% and 31% had prehypertensive and hypertensive ranges, respectively. Starting from gender, most individuals with normal BP range were females, at 81.8%. Pre-HTN and HTN were higher in males, at 51.7% and 58.1%, respectively.

Of the individuals in the most common group, 56% were prehypertensive, while 49.0% were hypertensive. Obese participants were more frequently listed under the categories pre-HTN and HTN, at 36.5% and 49%, respectively. Meanwhile, of the smokers, 36.5% were prehypertensive and 31.6% were hypertensive. A small proportion of employees who had heart diseases were prehypertensive and hypertensive, at 1.7% and 4.2%, respectively.

As shown in Table 2, gender, age, BMI, chronic diseases, HTN, and DM had a significant effect on random blood glucose measurements. Out of the 1000 participants, 3.5% and 5% were at the prediabetic and diabetic ranges, respectively. Out of the diabetic range individuals, 48% were also obese and 40% were overweight. Smoking, however, did not have a significant effect on random blood sugar ranges, with a P-value of 0.563, as only 17.1% of the smokers were prediabetic and 32% of the smokers were diabetic.

Table 3 shows the prevalence of controlled and uncontrolled readings of previously known cases of HTN and DM. Almost 38.6% of the individuals with HTN had it controlled, while 61.4% had uncontrolled HTN. However, 56.5% had controlled blood sugar, while 43.5% had uncontrolled ranges. Female individuals had a higher rate of uncontrolled HTN than male individuals, but males had a higher rate of uncontrolled DM. Of the uncontrolled HTN population, 49% belonged to the 31-40 year old age group, while 42% of uncontrolled DM ranges were 41-50 years old (Table 4). This was calculated according to their answers regarding their awareness of having or not having any chronic disease, including DM and HTN, and their measurements.

**Table 3:** Prevalence of control of hypertension and diabetes

	Controlled	Uncontrolled
Hypertension	27 (38.6%)	43 (61.4%)
Diabetes mellitus	39 (56.5%)	30 (43.5%)

**Table 4:** Control rate of hypertension and diabetes

	Hypertension		Diabetes	
	Controlled	Uncontrolled	Controlled	Uncontrolled
Gender				
Female	505 (73.2%)	130 (41.9%)	609 (64.1%)	26 (52%)
Male	185 (26.8%)	180 (58.1%)	341 (35.9%)	24 (48%)
Age (years)				
21–30	168 (24.3%)	48 (15.5%)	213 (22.4%)	3 (6%)
31–40	376 (54.5%)	152 (49%)	513 (54%)	15 (30%)
41–50	119 (17.2%)	77 (24.8%)	175 (18.4%)	21 (42%)
51–60	23 (3.3%)	30 (9.7%)	42 (4.4%)	11 (22%)
Above 60	4 (0.6%)	3 (1%)	7 (0.7%)	0 (0%)

Although some employees stated that they were free from any chronic diseases, they still had irregular readings. Regarding BP, 15.2% and 28.1% were unaware that they fall under the prehypertensive and hypertensive ranges, respectively. Nevertheless, 48.6% and 78.0% were unaware of being either prediabetic or diabetic, respectively.

Furthermore, 35% of the hypertensive participants were either prediabetic or diabetic, but they were unaware of it, while 20% of the diabetic employees were either prehypertensive or hypertensive (Table 1 and Table 2).

In addition, 66% were diagnosed with DM and had HTN (Table 5). The descriptive data are shown in Table 6.

**Table 5:** Prevalence of diabetes with hypertension

		Random blood glucose						
			Normal		Prediabetes		Diabetes	
Blood pressure	Normal		491 (53.7%)		10 (28.6%)		11 (22%)	
	Prehypertension		167 (18.3%)		5 (14.3%)		6 (12%)	
	Hypertension		257 (28.1%)		20 (57.1%)		33 (66%)	

**Table 6:** Descriptive statistics of patients SD, standard deviation

Descriptive statistics		
	Mean	SD
Age	36.83	7.928
Gender	1.37	0.482
Weight	74.9957	19.44406
Height	161.76	10.705
Body mass index	28.6829	7.61492
Blood pressure	1.8	0.884
Random blood glucose	1.14	0.466
Smoker (cigarette – shisha)	0.29	0.465
Chronic disease (none)	0.83	0.374
Chronic disease (dyslipidemia)	0.06	0.243
Chronic disease (hypertension)	0.07	0.255
Chronic disease (diabetes mellitus)	0.07	0.254

## Discussion

Saudi Arabia, like other regions worldwide, is facing a constant increase in the prevalence of both HTN and DM [12], which contribute greatly to the increase in CVD-related morbidity and mortality. A study performed on the burden of CVD in the Eastern Mediterranean region affirmed that patients with DM are four times more likely to develop CVD, compared to the general population, and are five times more likely to die due to it [5]. The explanation for this changing trend could be attributed to various risk factors. For instance, age, chronic diseases, and obesity contribute to the development of DM, while gender, age, and smoking habits contribute to developing HTN. This finding is consistent with that in another research completed in Qatar on the epidemiology HTN and its associated risk factors, stating that the independent risk factors of HTN include female gender, sedentary lifestyle, and advanced age [27].

The study objective was to examine the prevalence of DMs and HTN among the university employees, as these embody a major public health dilemma, along with careful analysis of the risk determinants. We also tested how screening at an early stage could contribute to lowering disease progression; calibrating the number of people affected by both DM and HTN is critical to allocate resources and plan for the future.

During the study, many adults were oblivious to their health condition. The total proportion of people who were not aware that they have DM or HTN was 8.5% even after reporting that they are free from any health condition; unawareness of one’s health condition is a contributing factor to many health challenges. A study carried out in Harjavalta and Kokemäki, Finland, concluded that, among 2676 people associated with the risk of CVD, 34.5% were unaware of their health status and were officially diagnosed with HTN [28]. Preliminary studies have proven that patient recognition and understanding of either HTN or DM is a prerequisite for prevention [8].

A study performed in Jeddah, Saudi Arabia, reported that the increase in the prevalence of DM increases with aging, which is also in line with the observation in this study, as 42% of diabetics were aged between 41 and 50 years old, while 40% of them between 31 and 40 years old were prediabetics [10].

The high proportion of DM and HTN individuals with a BMI of >30 kg/m^2^ in our sample accounted for 33.5% of a sample of 1000 participants. This demonstrates how obesity could be the leading causative factor for many chronic illnesses.

Studies have proven that gender hormones contribute a major role in the development of HTN, as to why gender-related differences exist [11]. In our population, the prevalence of HTN was higher in males. In 2018, the College of Medicine at Qassim University in Qassim, Saudi Arabia, analyzed a sample of 130 medical students and documented that 18.5% of the male candidates experienced HTN compared with that of only 5.3% of female candidates who had HTN [25], thereby justifying the role that androgens play to adjust BP between the two genders [24]. In contrast, a previous study conducted on 6024 patients with DM in Saudi Arabia by Dr. Alqurashi stated that the prevalence of DM is higher in males [9], which is not the case in this study, where the prevalence of DM is higher in females. This may be due to the higher ratio of female to male employees.

A study performed worldwide on 4.4 million participants stated that the prevalence of DM in males had doubled since 1980, while that in females increased by 60%. However, our study showed no significant difference between the two genders [10]. Furthermore, the Imperial College London conducted a pooled analysis consisting of 19.1 million participants regarding the prevalence of elevated BP among different counties where studies have been conducted to measure participants’ SBP and DBP levels.

The previously mentioned study asserted that the age-stratified mean SBP of participants aged 18 years and older persisted without a change from 1975 to 2015 in males (126.6 mmHg [95% CI: 124.0-129.3] in 1975 and 127 mmHg [95% CI: 125.7-128.3] in 2015, which is an increase of 0.07 mmHg per decade [95% CI: −0.59 to 0.74]), while it mildly decreased in females (123.9 mmHg [95% CI: 121.3-126.6] in 1975 and 122.3 mmHg [95% CI: 121.0-123.6] in 2015, which is a decrease of 0.47 mmHg per decade [95% CI: −0.20 to 1.15], along with similar trends in the mean DBP in both males (78.7 mmHg) and females (76.7 mmHg) in 2015. High income in the Asia Pacific has led to the greatest decrease in mean SBP, while high income in the western superregion has led to the greatest decrease in mean DBP in each decade [13].

A systematic review was conducted to compute the global burden of disease in the year 2019 and reported increased exposure to drug use, air pollution, lead poisoning, elevated fasting plasma glucose, and high BMI. In the same year, an elevated SBP was among the greatest risk factors attributed to mortality, followed by tobacco as a substance of varied use. There was a significant variance in the risk factor burden in relation to both age group and setting [29]. One of the aims of the training program was to collect employees’ data and to refer any high-risk individuals to the staff clinic at the university hospital.

The present study expanded this proposition to what seems to be an indirect way of screening that showed 22% of employees with DM and 28.1% hypertensive employees are unaware of their condition. A study conducted in the USA confirmed the importance of screening and its role in enhancing the quality of life of an individual by conducting a cohort study where 100,000 participants computed the health impact and calculated that 15,600 quality-adjusted life years (QALYs) would result from screening for HTN [30]. A study carried out in Fortaleza, Brazil, demonstrated proof that early screening and prevention play a crucial role in quality of life improvement [19].

A cross-sectional study conducted in Riyadh, Saudi Arabia, in 2014 on the quality of life of patients with type 2 DM reported that, when compared to the healthy general population, patients with DM had a moderate decrease in HRQoL.

A study was conducted to analyze the impact of smoking on DM and indicated a positive strong correlation between smoking and the development of DM and its complications. This was also true in our own study, as 32% of the smokers had DM. Smoking cessation is an accessible and cost-effective way to prevent the development of DM and its complications [7].

Of the 31% of the patients with hypertension, the results showed that merely 3.9% managed to have good control over their BP. In 2017, the University of Oxford conducted a meta-analysis that provided righteous knowledge about the effectiveness of self-monitoring BP while also including other interventions necessary to surpass the patient’s lifestyle, which contributes to disease control [26]. The meta-analysis pointed out the correlation between self-monitoring and reduced DBP in a 12-month follow-up (DBP: −1.5 mmHg [95% CI: −2.2 to −0.8 mmHg]) [23]. In line with the results of this study, another meta-analysis calculated the weighted mean difference (WMD) to prove that, in self-monitored BP, there is a slight but significant reduction in the BP readings (SBP: −3.82 mmHg [95% CI: −5.61 to −2.03], DBP: −1.45 mmHg [95% CI: −1.95 to −0.94]). Self-monitoring increased the chance of meeting office BP targets (12 randomized controlled trials, 13 comparisons, 2260 patients, relative risk = 1.09 [95% CI: 1.02-1.16]) [22].

In this study, only 4.1% of the sample population had controlled DM, while 60% of the individuals’ blood glucose was poorly controlled; a high proportion of patients experiencing DM in Saudi Arabia had poor control over their blood glucose. This can lead to diverse long-term complications, which will, ultimately, lead to poor quality of life [18]. Therefore, patient education and understanding are important to achieve good compliance and prevent serious complications.

Hence, the establishment of such a policy would be of good use for upcoming studies [26]. The results include information about the prevalence of DM and HTN, not about social insurance policies. The cross-sectional study based in Korea in 2018 incorporated central obesity and calculated it to be in 20% of their 1,025,340 sample population based on BMI readings. Their total prevalence rates have been calculated to be 25.8% for HTN [95% CI: 25.5-26], 10.2% for DM [95% CI: 10.1-10.4], and 16.6% for dyslipidemia [95% CI: 16.4-16.8]. Most participants included in the study have been aware of their diagnoses, except that for dyslipidemia, in which only 24.1% knew about their health condition.

Like any other research, this study has limitations, including negligence to detect central obesity, physical activity, and dietary habits, which are cardinal risk factors seen in the diagnosis of DM and HTN, as well as the inability to acknowledge the prevalence of hyperlipidemia, which is considered one of the chronic conditions causing serious CVD, due to the lack of laboratory resources. As the training program conducted elective workshops for many departments at university campuses, the sample size did not accompany an equal number of males and females to identify/address gender-related/age-related or other specific risk factors.

## Conclusions

This study measured the prevalence of DM and HTN among employees of KAU. The high prevalence of DM and HTN across our community, lack of early screening, unawareness of one’s health condition, poor disease control, obesity, smoking, and high physical inactivity reflect the importance of paying more attention to this matter. These individuals face multiple social, health, and financial struggles, and patient education is important to achieve good compliance and prevent serious complications. To conclude with, we hope that more awareness should be raised among the target population. This will result in a drastic improvement of their quality of life, reduce unnecessary financial burdens, and shift their focus to a better lifestyle in the near future.

As the study aims to detect HTN and DM at an early stage, it is anticipated that future practice would be directed on prevention rather than treatment, particularly when it comes to frequent chronic diseases. Adjusting a patient’s lifestyle, smoking cessation, obesity control, and risk factors would impact each patient’s quality of life drastically. Nevertheless, a screening clinic is best to achieve this result as well as for the safety of the employees. Moreover, employees should mandatorily undergo annual screening, and they must be referred to primary healthcare centers. There is insufficient evidence on public screening and awareness; therefore, more research should be conducted on this topic. Moreover, well-being can affect an employee’s quality of life and performance. More research should be expanded into the relationship between chronic diseases and an employer’s attendance and productivity.
